# Application of real-time PCR to quantify hepatitis B virus DNA in chronic carriers in The Gambia

**DOI:** 10.1186/1743-422X-3-23

**Published:** 2006-04-04

**Authors:** Maimuna E Mendy, Steve Kaye, Marianne van der Sande, Pura Rayco-Solon, Pauline A Waight, Deborah Shipton, Dorka Awi, Paul Snell, Hilton Whittle, Samuel J McConkey

**Affiliations:** 1 Medical Research Council, Atlantic Boulevard, Fajara, P O Box 273, Banjul, The Gambia; 2 Viral Disease programme, Medical Research Council, Atlantic Boulevard, Fajara, P O Box 273, Banjul, The Gambia; 3 Imperial college, London, UK; 4 RIVM, Bithoven, The Netherlands; 5 Nutrition centre of the Philippines, Philippines; 6 National Protection Agency, Collindale, London, UK; 7 Royal College of Surgeons in Ireland, Dublin, Ireland

## Abstract

**Background/Aim:**

The study aimed at developing a real-time quantitative PCR assay to monitor HBV serum virus load of chronic carriers enrolled in therapeutic trials.

**Method:**

Quantitative real-time PCR assay was carried out using SYBR-Green signal detection and primers specific to the S gene. Thermal cycling was performed in an ABi 5700 sequence detection system. The assay was calibrated against an international HBV DNA standard and inter- and intra-assay reproducibility determined. Levels of viral load were monitored for 1-year in lamivudine treated carriers. Correlation between HBV DNA levels and HBeAg sero-status was determined in untreated carriers.

**Results:**

The qPCR assay showed good intra- and inter-assay reproducibility over a wide dynamic range (1.5 × 10^3 ^to 1.5 × 10^8 ^copies/mL) and correlated well with those from a commercial assay (r = 0.91, (p < 0.001). Viral load levels dropped dramatically but temporarily during and after a short course of lamivudine therapy. HBV DNA was a more reliable indicator of the presence of virus than HBe antigen and was detected in 77.0% (161/209) of HBeAg negative and in all HBeAg positive carriers.

**Conclusion:**

This method is reliable, accurate, and reproducible. HBV DNA Quantification by qPCR can be used to monitor the efficacy of HBV therapy and useful in understanding the natural history of HBV in an endemic area.

## Introduction

Hepatitis B virus (HBV) is the leading cause of viral hepatitis in humans worldwide. Currently over two billion people have evidence of previous HBV infection and 350 million have become chronic carriers of the virus, 60 million of them residing in Africa [1]. In the Gambia, where HBV is endemic, the prevalence of chronic infection is 10–15% of the adult population [2,3]. Chronically infected carriers have a high risk of developing liver damage and hepatocellular carcinoma (HCC) and liver cancer is the commonest cause of death in adult males in The Gambia [4]. Detection of serological markers is the mainstay of diagnosis of HBV infection and the most reliable marker of HBV carriage is HBV surface antigen (HBsAg) in serum. HBV e antigen (HBeAg) is generally used as secondary marker to indicate high levels of virus in the blood. The minority of chronic HBV carriers in whom HBeAg can be detected have a particularly high risk of progressive liver disease and end stage liver failure [5]. The monitoring of hepatitis B virus DNA in serum is as important as serological markers in predicting the clinical outcome of infection. More recently molecular diagnostic methods have been used to quantify the levels of HBV DNA in serum as a marker of viral replicative activity [6]. The detection and quantification of HBV DNA is reported to have prognostic value for the outcomes of acute and chronic HBV infections [7,8]. Quantification of HBV DNA may be a more useful measure than HBeAg as genetic variants of HBV may continue to replicate at high level without secreting HBeAg. Quantification of HBV DNA can be useful to assess the efficacy of antiviral therapy as a more direct method of detecting viral replication than HBV serologic markers [9,10]. The clinical management of HBV could be improved by the use of accurate quantification of virus load as a measure of replication of HBV in patients with chronic liver disease. Prior to the development of the polymerase chain reaction (PCR) a number of hybridisation methodologies were used to monitor HBV DNA levels [11-14]. The introduction of PCR-based methods has resulted in a large increase in the sensitivity of HBV DNA detection and commercialisation of PCR-based methods (e.g. HBV Monitor, Roche Diagnostic Systems) has lead to widespread adoption of the methodology [15]. More recently the development of real time PCR methodology has further improved the ease with which HBV DNA levels can be monitored and has increased the range over which such levels can be accurately quantified [16,17].

We describe the development and validation of a quantitative PCR (qPCR) method to measure the concentration of HBV DNA in serum. The assay is based on the specific amplification of HBV DNA using primers targeted to the S-gene and detection in real-time with SYBR Green dye.

The specificity, reproducibility and detection limit of the assay was examined. The assay was used to monitor HBV DNA levels in patients on lamivudine therapy.

Viral load in HBeAg-positive and HBeAg-negative asymptomatic HBV carriers was measured to assess the relationship between serologic markers and levels of HBV DNA.

## Material and methods

### Study subjects

Two groups of asymptomatic HBV carriers were included in the study. The first consisted of 22 male, asymptomatic HBV carriers aged between 15 and 25 years recruited to a therapeutic vaccine trial. As part of this trial some subjects were randomised to receive the antiviral drug lamivudine (GlaxoSmithKline). Fifteen subjects received a 98- day course of lamivudine therapy alone and seven were monitored as untreated controls. The volunteers were followed for a period of 255 days; blood samples were collected at baseline then on 28, 56, 77, 98, 161, 245 and 329 days after the baseline visit. The Gambian Ethics Committee reviewed and approved both studies.

The second group consisted of 318 HBV chronic carriers aged between 1 and 73 years recruited during 1983 and 2003 community survey conducted in three rural villages in The Gambia. An infant vaccination programme started in these villages in 1984 so the majority of the HBV carriers were over 20 years. Levels of viral load was compared with HB sero-status.

### Real-time quantitative PCR for HBV DNA

DNA was extracted from 200 μL of serum using the QIAamp DNA Mini Kit (Qiagen, Hilden, Germany) according to the manufacturer's instructions. DNA was eluted into 100 μL nuclease-free water and 5 μl added to a 25 μl PCR reaction mixture.

The reaction was carried out using a commercial SYBR-Green reaction mix (Qiagen, Hilden, Germany). The kit contains HotStarTaq polymerase which is included to avoid false positives in the quantitative PCR. The primer sequences were 5'-GTG TCT GCG GCG TTT TAT CA (sense) and 5' GAC AAA CGG GCA ACA TAC CTT (antisense) designed to amplify a 98 base pair product from positions 379 to 476 of the HBV genome [18]. Thermal cycling was performed in an ABi 5700 sequence detection system (PE Applied Biosystems, Warrington, UK). Reaction conditions were: 95°C for 15 minutes followed by 40 cycles of 94°C for 15 seconds, 55°C for 30 seconds and 72°C for 30 seconds. A four point standard curve (1.5 × 10^8^copies per millilitre (cpm), 1.5 × 10^6^cpm, 1.5 × 10^4^cpm, 1.5 × 10^2^cpm) was generated from a high titre plasma donation quantified by end point dilution PCR. The calibration of this standard was confirmed by comparison with an International HBV DNA standard, (97/746) (NIBSC, Potters Bar, UK). Test samples falling above the top of the standard curve were re-assayed at a dilution of 1:100. Each test run included positive and negative controls. The performance of the assay was evaluated by comparison with a commercial assay (HBV Monitor, Roche Molecular Systems, Inc., Branchburg, NJ 08876 USA) performed according to the manufacturer's instructions.

### Serology

Subjects were tested for HBV core antibody (anti-HBc) and if indicated HBV surface antigen (HBsAg) and HBV e antigen (HBeAg). Anti-HBc was measured by radio-immunoassay (RIA) (Sorin Biochemica, Saluggia, Italy) AB-COREK test kit. Samples, which were anti-HBc positive, were tested for HBsAg by reverse passive haemagglutination assay (RPHA) (Wellcotest^®^, Murex Diagnostics, Dartford, UK) and or by Determine™ HBsAg (Abbott Laboratories), an immunochromatographic assay. HBsAg positive subjects were tested for HBeAg using an enzyme immunoassay (EIA) (Equipar Diagnostici, Saronno (Va), Italy).

### Data management

The data obtained in the ABi real time machine after the PCR amplification and quantification of DNA was exported as an Excel spreadsheet into an Access database designed for the study. The viral results were merged with HBV serological results prior to data analysis.

## Results

### Performance of the real-time qPCR

The performance of the new qPCR assay was examined by determining the sensitivity, specificity, inter- and intra-assay variability. HBV DNA standard was obtained by end point dilution assay then calibrated with known standard (97/746, NIBSC, Potters Bar, UK).

To determine the HBV DNA concentration of the top standard serial half-log dilutions from 1:10 to 1:10^10 ^were prepared from an HBeAg-positive plasma. The plasma was diluted in HBV DNA-negative EDTA plasma. DNA was extracted and amplified in quadruplicate reactions. The end point, the dilution resulting in a mixture of positive and negative reactions, was obtained at a dilution of 1:10^7.5 ^at which three of four reactions were positive. HBV DNA concentration was calculated as 4.3 × 10^9.0 ^copies/ml. This value was adjusted to 1.5 × 10^8 ^after direct comparison to the International HBV DNA standard.

The detection limit of the assay was 2.6 × 10^2 ^DNA copies per mL when used to test a serial dilution of a 2.6 × 10^6 ^DNA standard. The assay was 100% specific when tested against HBV seronegative sera from ten subjects. To evaluate the reproducibility of the assay five samples were tested in triplicate on three different occasions. Variability between the triplicate samples assayed in the same run was 3.06% and variability between mean results of triplicates samples in different runs was 2.35%. Overall variability = 3.03%. The coefficient of variation obtained from intra-and inter assay was 1.08 and 1.72 respectively (Tables 1 and 2).

**Table 1:** Intra assay variability of the newly developed qPCR assay

	DNA (copies DNA per mL) in experiment					
Sample ID	Experiment 1	Experiment 2	Experiment 3	Average	SD	CV (%)

MVA 300	2.6 × 10^6^	2.5 × 10^6^	1.6 × 10^6^	2.3 × 10^6^	0.104	1.60
MVA 307	1.9 × 10^9^	4.5 × 10^9^	1.7 × 10^9^	2.7 × 10^9^	0.191	2.00
MVA 313	1.2 × 10^8^	1.2 × 10^8^	1.2 × 10^8^	1.2 × 10^8^	0.004	0.50
MVA 315	2.5 × 10^6^	2.2 × 10^6^	1.9 × 10^6^	2.2 × 10^6^	0.046	0.70
T4040	4.7 × 10^3^	4.9 × 10^3^	6.4 × 10^3^	5.4 × 10^3^	0.060	1.60
Negative control	not detected	not detected	not detected			

The six samples represented in the table include five positive samples (MVA 300, MVA 307, MVA 313, MVA 315) and one negative sample (negative for HBsAg). The samples were tested three times in the same assay.

**Table 2:** Inter assay variability of the newly developed qPCR assay

	DNA (copies per mL) on different days					
Sample ID	Day 1	Day 2	Day 3	Average	SD	CV (%)

MVA300	2.7 × 10^6^	1.4 × 10^6^	2.7 × 10^6^	2.3 × 10^6^	0.19	3.0
MVA307	2.1 × 10^9^	4.2 × 10^9^	1.5 × 10^9^	2.6 × 10^6^	0.26	2.70
MVA313	1.4 x10^8^	9.8 × 10^7^	1.3 × 10^8^	1.2 × 10^8^	0.09	1.10
MVA315	1.3 × 10^6^	3.3 × 10^6^	2.0 x10^6^	2.2 × 10^6^	0.01	1.20
T4040	5.6 × 10^3^	7.2 × 10^3^	4.3 × 10^3^	5.7 × 10^3^	0.23	0.62
Negative control	DNA not detected	DNA not detected	DNA not detected	DNA not detected		

The six samples represented in the table include five positive samples (MVA 300, MVA 307, MVA 313, MVA 315) and one negative sample (negative for HBsAg). The samples were tested on three different days.

**Figure 1:**
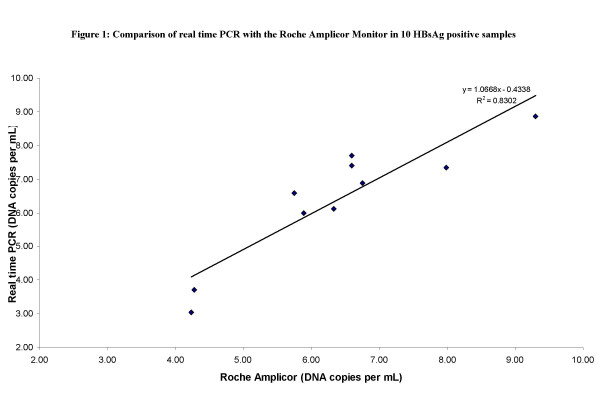
**Comparison of real time PCR with the Roche Amplicor Monitor in 10 HBsAg positive samples**. The log difference between the two assays was less than 1.0 over the dynamic range of 1.1 × 10^3 ^to 2.0 × 10^9^.

To further validate the performance of the in-house method it was compared to a commercial quantitative PCR method (Roche Amplicor Monitor, Inc., Branchburg, NJ 08876 USA). Ten samples were assayed in the two assays and the results are shown in Figure 1. Over a range of 1.1 x10^3 ^to 2.0 × 10^9 ^DNA copies per mL the difference between the two assays was less than 1.0 log. The correlation between the log results of the two assays was high (r = 0.91, p < 0.001).

### Correlation of HBV DNA loads and HBeAg in asymptomatic carriers

One hundred and eight (34%) of the 318 HBsAg positive carriers had detectable HBeAg and the proportion decreased with age (Chi squared test for trend p < 0.0001). All HBeAg positive carriers tested positive for HBV DNA compared to 164 (78%) of the HBeAg negative carriers (Table 3.).

**Table 3:** Viral load in relation to age group of carriers and HBeAg status

	HBeAg Positive subjects		*HBeAg Negative carriers*		
Age group (Yrs)	DNA positive (%)	Viral load (GM)	DNA positive (%)	Viral load (GM)	Total DNA positive in all carriers

<5	50/50 (100%)	3.2 × 10^8^	9/9 (100%)	4.3 × 10^4^	59/69 (85.5%)
5–9	39/39 (100%)	3.8 × 10^8^	15/21 (71.4%)	9.3 × 10^4^	54/63(85.7%)
10–19	5/5 (100%)	1.7 × 10^9^	11/13 (84.6%)	1.3 × 10^4^	16/18 (88.9%)
20–29	11/11 (100%)	8.1 X10^7^	71/91 (78.0%)	4.9 X10^3^	82/102 (80%)
30–39	3/3 (100%)	5.5 X10^7^	23/33 (69.6%)	2.6 X10^3^	26/36 (72%)
>40	1/1 (100%)	4.6 X10^2^	32/42 (76.1%)	6.02 X10^3^	33/43 (77%)
Total	109/109 (100%)		161/209 (77.0%)		270

As it is most likely that HBV infection occurs in childhood in the Gambia, the age of the carriers is likely to represents the length of HBV carriage.

The geometric mean concentration of HBV DNA in HBeAg-positive carriers was 4.6 log10 copies per ml higher than in HBeAg-negative carriers (8.7 log10 copies per ml vs. 4.1 log10 copies per ml, p < 0.0001). The viral load was lower with increasing age in both HBeAg -positive and HBeAg negative carriers (p = 0.032, R = 0.207 for HBeAg positive carriers and p <0.0001, R = 0.275 for HBeAg negative carriers).

### Monitoring of HBV DNA loads in subjects receiving lamivudine therapy

**Figure 2:**
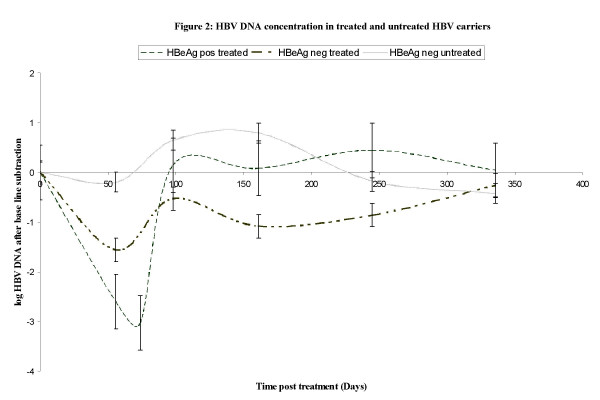
**HBV DNA concentration in treated and untreated HBV carriers**. The three groups consisted of HBeAg positive carriers treated with LMV (7), HBeAg negative carriers treated with LMV (8) and HBeAg positive carriers untreated and did not receive any LMV (7). Patients were tested on day 56, 98, 161, 245 and 329 after LMV treatment. HBeAg positive carriers had an additional test on day 77. The horizontal lines indicate error bars.

The level of HBV DNA was measured in 16 asymptomatic carriers (eight HBeAg positive and eight HBeAg negative) on daily dose of 100 mg lamivudine for 98 days. HBV DNA was measured at day 56, 98, 161, 245 and 329 after the start of treatment with LMV. For comparison seven asymptomatic untreated HBeAg negative carriers were tested for HBV DNA. The baseline characteristics of the two HBeAg negative groups (treated and untreated) were similar (data not shown). None of the carriers had totally cleared the virus as a result of LMV treatment however HBV DNA decreased in patients on LMV and not in the untreated patients. Reduction in viral load was higher in HBeAg positive carriers than in HBeAg negative carriers, 3.2 log compared to 1.5 respectively (Figure 2). Viral load level returned to base line levels soon after the withdrawal of LMV and this was more noticeable in the HBeAg positive group. There was subsequent rebound of viral load at the withdrawal of LMV therapy. Fluctuation of level of viral load was observed in the untreated group

## Discussion

We have described the development and validation of a real-time polymerase chain reaction (PCR) method based on SYBR-Green for, measuring HBV DNA in serum and plasma. We have demonstrated that the method had good specificity, reproducibility and sensitivity and gave comparable results to those obtained with a commercial assay. The primers used in this study have been shown to produce similar amplification/detection efficiency when used to test samples of genotypes A and G [18]. The method described had a lower threshold of detection than the commercial assay with which it was compared [19]. In general real-time PCR methodology is robust and easy to perform and avoids many of the potential contamination pitfalls that are associated with gel-based and hybridization-based post-PCR detection methods.

The assay was used to assess the virological response to short-term treatment with anti-viral medication. As for HIV there is a large unmet need for treatment of chronic HBV infection in sub-Saharan Africa. This is concurrent with high morbidity and mortality from liver cancer and cirrhosis. As antiviral therapies become more affordable there will be requirement for virological assessment of the successes of the treatment [20]. The assay described here could have such a role. The lack of total clearance of HBV DNA is most likely due to short-term treatment. Sustained response after discontinuation of treatment was uncommon in the carriers in this study and occurs in only 10–15% of patients treated for years with LMV [21]. Drug resistance in most cases after long-term therapy with LMV has created the need for alternative form of treatment for viral load reduction such as pegylated interferon [22,23]. Although LMV treatment can be effective in some cases, low response rate to treatment is evident in HBeAg negative patients [24].

The new assay was used to describe the course of viraemia in chronically infected people in The Gambia. The results of the cross-sectional study suggest that the characteristics of chronic HBV infection changes as people get older, or have the infection for a longer time. Fewer older people had HBeAg in the blood, suggesting that this is lost over time in this group. The lower viral loads levels in those who are HBeAg negative suggest that the host's control of the virus replication is stronger and more efficient in those who are older, or who have had the infection for longer. This group, who have mostly been infected early in life, are immunotolerant of the virus and often show little or no clinical hepatitis [2]. There is evidence that infected carriers are partly controlling HBV viral load, even before they lose HBeAg from the blood. As shown in this study there is a significant decline in viral load with age in both HBeAg positive and HBeAg negative carriers.

The results of this study suggest that despite the immunotolerance as they get older HBV carriers appear to clear HBeAg and partially control viral replication. It is unclear why the immune response may be more effective in those who are older.

The cross-sectional component of this study shows that a new rapid, robust, repeatable quantitative PCR assay for HBV viral load can provide a useful tool to understand the complex interactions between the HBV virus and the infected host. This could be developed through its application in a longitudinal study, ideally incorporating simultaneous measures of the host immune response. The longitudinal study of a group of chronic HBV carriers who spontaneously loose HBeAg or seroconvert from HBsAg to anti-HBs could be pivotal in elucidating the immunological mechanisms, which affect the control of virus, which these changes entail. This may be necessary prelude for the development of effective immunomodulatory interventions for the 350 millions of chronically infected individuals.
